# Supplementary figures and images for: The dynamic response of human lungs due to underwater shock wave exposure
Source: PLoS One. 2024 May 15;19(5):e0303325. doi: 10.1371/journal.pone.0303325 (PMC11095682; doi:10.1371/journal.pone.0303325)

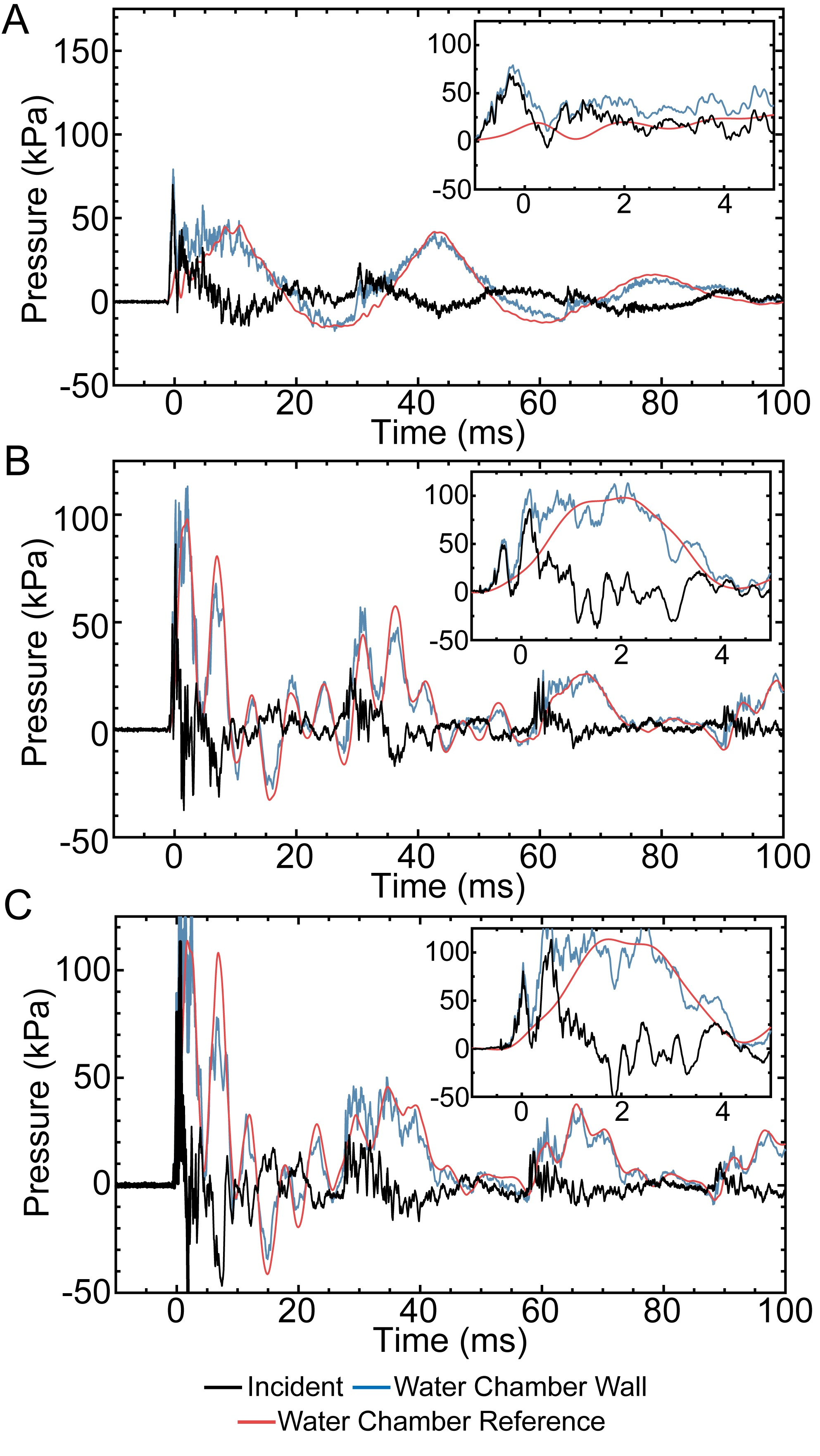

Supplement: S1 Fig — The incident pressure (black) was computed by subtracting a filtered reference pressure measurement (red) from the pressure measurement made at the wall closest to the diaphram (green). (TIF) [file pone.0303325.s001.tif]

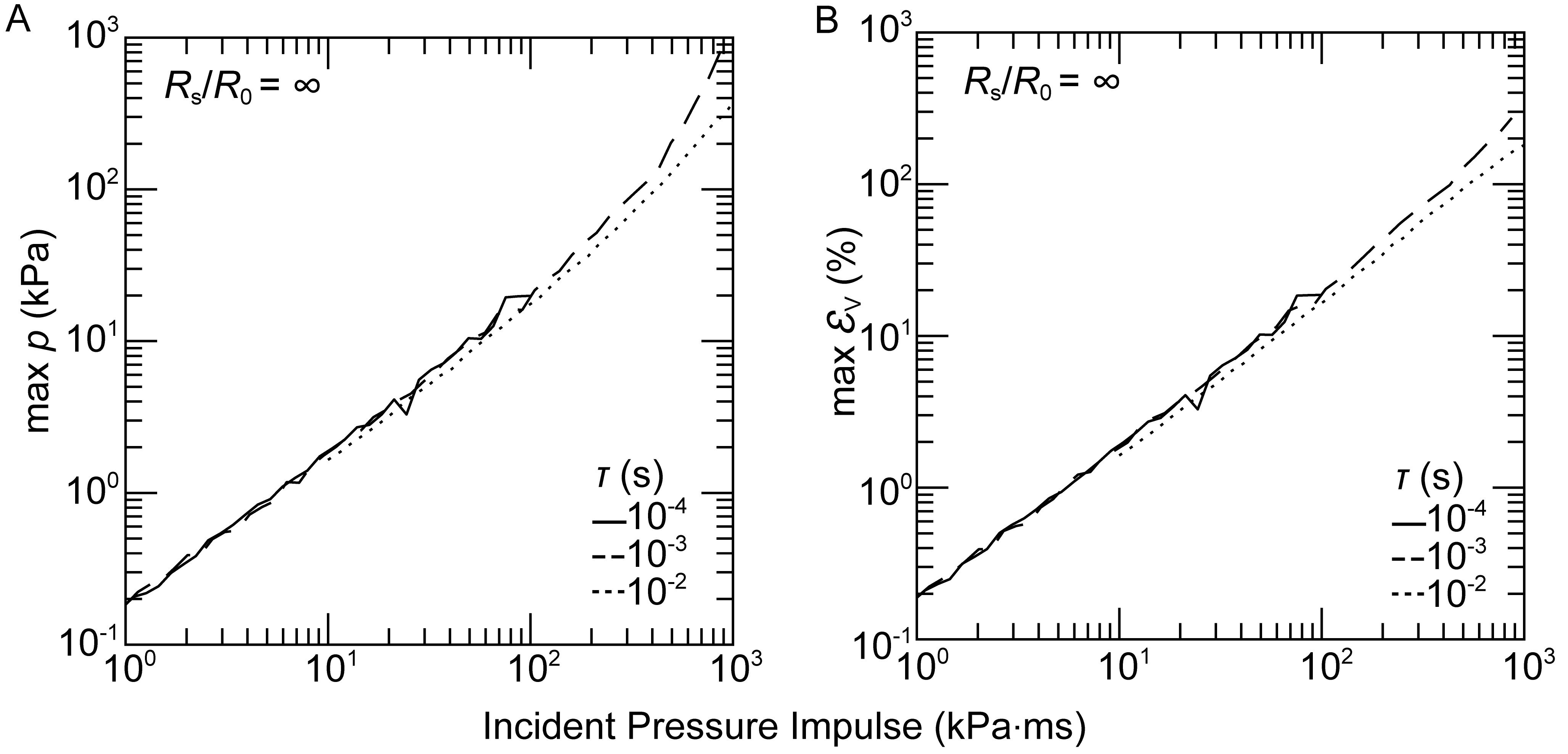

Supplement: S2 Fig — Analytical solution of the Rayleigh-Plesset equation of a spherical gas bubble with initial radius R0 within a unconstrained spherical water chamber of radius RS = ∞. Maximum (A) bubble pressure p and (B) volumetric strain εV for increasing values of pressure impulse for incident pressure durations of τ = 10−4 s (solid), 10−3 s (dashed), and 10−2 s (dotted). (TIF) [file pone.0303325.s002.tif]
